# Tinnitus evaluation: relationship between pitch matching and loudness, visual analog scale and tinnitus handicap inventory^☆^

**DOI:** 10.1016/j.bjorl.2018.05.006

**Published:** 2018-06-21

**Authors:** Islan da Penha Nascimento, Anna Alice Almeida, José Diniz, Mariana Lopes Martins, Thaís Mendonça Maia Wanderley Cruz de Freitas, Marine Raquel Diniz da Rosa

**Affiliations:** a Universidade Federal da Paraíba (UFPB), Hospital Universitário Lauro Wanderley (HULW), João Pessoa, PB, Brazil; b Universidade Federal da Paraíba (UFPB), Departamento de Fonoaudiologia, João Pessoa, PB, Brazil; c Universidade Federal da Paraíba (UFPB)/Universidade Federal do Rio Grande do Norte (UFRN), Programa Associado de Pós-graduação em Fonoaudiologia (PPgFon), Brazil; d Universidade Federal da Paraíba (UFPB), Programa de Pós-graduação em Neurociência Cognitiva e Comportamento (PPGNeC), João Pessoa, PB, Brazil; e Universidade Federal da Paraíba (UFPB), Programa de Pós-graduação em Modelos de Decisão e Saúde (PPgMDS), João Pessoa, PB, Brazil; f Universidade Federal do Rio Grande do Norte (UFRN), Natal, RN, Brazil

**Keywords:** Tinnitus, Sound perception, Hearing, Audiology, Questionnaire, Zumbido, Percepção sonora, Audição, Audiologia, Questionário

## Abstract

**Introduction:**

Tinnitus is a subjective auditory symptom usually associated with a sound, even in the absence of external sound sources. Its diagnosis is complex, and some of the forms of measurement alone or in combination, include self-assessment questionnaires, such as the tinnitus handicap inventory, the visual analog scale and/or pitch and loudness matching.

**Objective:**

To analyze the correlation among three tinnitus measurement methods: tinnitus handicap inventory, visual analog scale and pitch and loudness matching.

**Methods:**

The study consisted of 148 patients complaining of chronic tinnitus. An otorhinolaryngological evaluation, anamnesis directed to tinnitus, audiometry (pure tone and speech), imitanciometry, tinnitus handicap inventory, visual analog scale, and pitch and loudness matching were performed. The study was registered in the Ethics Committee of the Institution with no. 0129/12.

**Results:**

Regarding the frequency of tinnitus handicap inventory responses, a higher occurrence of the mild degree was observed. An average of 6 points was observed on the visual analog scale. The mean loudness matching in the right ear was 20 dBNS, and in the left ear was 17 dBNS. As for the type of stimulus, the most found was continuous pure tone. The frequency of the pitch sensation was 6000 Hz in the largest number of cases. Regarding the measures of tinnitus handicap inventory and the visual analogical scale, a significant correlation was observed, and as one value increases the other also increases. Pitch and loudness matching and the visual analogical scale results are also significant.

**Conclusion:**

There was a significant correlation between the values measured by the tinnitus handicap inventory, visual analogical scale (annoyance) and loudness matching in the evaluation of tinnitus. The selection of any one of the three evaluative methods for tinnitus investigation provides different dimensions of the tinnitus and complements the others.

## Resumo

**Introdução:**

O zumbido é um sintoma auditivo de natureza subjetivo, normalmente associado a um som, mesmo na ausência de fontes sonoras externas. Apresenta diagnóstico complexo. Uma das formas de mensuração é o uso, isolado ou em conjunto, de questionários de autoavaliação, como o *Tinnitus Handicap Inventory*, a escala visual analógica e o exame de acufenometria.

**Objetivo:**

Analisar a correlação entre os métodos de mensuração do zumbido: *Tinnitus Handicap Inventory*, escala visual analógica e acufenometria.

**Método:**

Participaram do estudo 148 pacientes com queixa de zumbido crônico. Realizou-se avaliação otorrinolaringológica, anamnese direcionada ao zumbido, audiometria (tonal e vocal), imitanciometria, *Tinnitus Handicap Inventory*, escala visual analógica e acufenometria. O estudo foi registrado no comitê de ética da instituição, com n° 0129/12.

**Resultados:**

Em relação à frequência das respostas do *Tinnitus Handicap Inventory*, foi observada maior ocorrência do grau leve. Observou-se uma média de 6 pontos na escala visual analógica. A média da sensação de intensidade medida na acufenometria na orelha direita foi de 20 dBNS e na orelha esquerda foi de 17 dBNS. Quanto ao tipo de estímulo, o mais encontrado foi o tom puro contínuo. A sensação de frequência, no maior número de casos, foi de 6.000 Hz. Entre as medidas do *Tinnitus Handicap Inventory* e da escala visual analógica, observou-se correlação significante. À medida que um valor aumenta o outro também aumenta. Os resultados da acufenometria e da escala visual analógica também apresentam significância.

**Conclusão:**

Houve correlação significante entre os valores medidos pelo *Tinnitus Handicap Inventory*, escala visual analógica (incômodo) e acufenometria (*loudness*) na avaliação do zumbido. A escolha de um dos três métodos avaliativos para pesquisa do zumbido fornece dimensões diferentes do zumbido e se complementam.

## Introduction

Tinnitus is an auditory sensation in the absence of an external sound source.^1^, ^2^ With an estimated incidence of 10%–15% in the world population, most people experience a minimal impact on quality of life, but it is clinically significant for approximately 20% of those with chronic tinnitus.^3^ In a recent study with the population of the city of São Paulo, a prevalence of 22% was found, and 64% of affected individuals felt uncomfortable with the sound.^4^ More recent theories on the pathophysiology of tinnitus point to the existence of increased or nonsynchronized neuronal discharges in the central auditory pathways, initiated secondary to an auditory system dysfunction.^5^, ^6^

Among the several factors hindering the use of therapies for tinnitus is the lack of standardization of the Tinnitus Assessment and Measurement Methods (TAMM). The selected assessment method is expected to have the following characteristics: (1) brief, to be possible to use in clinical practice; (2) easy to apply and interpret; (3) covers various aspects of tinnitus; (4) is validated and reliable.^7^

Of the methods used to assess tinnitus, the tinnitus handicap inventory (THI), the measurement of psychoacoustic characteristics, and the evaluation of the handicap caused by tinnitus through the Visual Analog Scale have been the most used.^8^, ^9^

A recent study in the UK found that THI is the tinnitus questionnaire most commonly used by physicians in that country.^10^ It should be noted that even though it is one of the most used questionnaires, care should be taken with the sample and the way it will be applied.

The need for psychoacoustic measures to characterize tinnitus, also called tinnitometry, was first reported in 1931, but only with the invention of suitable electroacoustic equipment was it possible to measure the sensation of loudness and pitch of the sound that is perceived only by the patient.^11^, ^12^ Tinnitus characterization is complex because it is a subjective symptom; thus, only the patient can recreate a similar sound, which can often be multitonal and change loudness.

Subjective assessment of the discomfort from intense tinnitus can be performed using a visual analog scale (VAS).^13^ Although widely used, it has not yet been systematically validated for the evaluation of different aspects of tinnitus.^14^

The literature has not established any consensus that these three evaluative methods of tinnitus (THI, VAS, and loudness and pitch matching) have a correlation with each other.^15^ There are some authors who have found a correlation between psychoacoustic measures and subjective measures,^8^ while others did not.^16^ Thus, many scientific papers frequently use these three methods for evaluating patients with tinnitus.

The aim of the present study was to analyze the correlation among tinnitus measurement methods using THI, VAS (when assessing the grade of handicap) and loudness/pitch matching.

## Methodology

The present study is characterized as an observational, cross-sectional, quantitative study. It was approved by the Research Ethics Committee (according to report no. 0129/12), and all volunteers who participated in the study signed an Informed Consent term.

The study sample consisted of 148 volunteers who met the following eligibility criteria: age between 18 and 60 years; to have tinnitus (unilateral, bilateral or on the head), and low anxiety-trait (through the application of the State-Trait Anxiety Inventory/STAI).^17^

The sample calculation was based on a 95% confidence level and a sample error of 5%, taking into account that 28 million Brazilians, and 22% of the population of São Paulo report the symptom. Thus, 262 volunteers were estimated. In the service where research was carried out, 270 patients were evaluated; however, due to the absence of some data such as pitch and loudness matching, VAS, or THI scores, high anxiety and some refusing to participate in the study, 122 volunteers were excluded.

After signing the term of free prior and informed consent (FPIC), the volunteers were evaluated as follows: otorhinolaryngological evaluation; audiological and specific anamnesis for tinnitus; basic audiological evaluation (pure tone/speech audiometry and imitanciometry); pitch and loudness matching; tinnitus handicap inventory (THI) and visual analog scale (VAS).

The specific anamnesis for tinnitus is primarily aimed at collecting tinnitus data from the patient's perspective, such as: location: right ear, left ear, both ears or head; time through which tinnitus is present: days, weeks, months, or years; the onset of tinnitus: sudden, gradual, after exposure to noise or other; tinnitus type: continuous, pulsatile or intermittent; acoustic characteristics similar to sound heard: sound of whistle, rain, wheezing, cascade, bee/fly, or other; period when tinnitus is heard: in the morning, in the afternoon, at night, all the time, when lying down, or other; sound loudness: high, medium or low; relationship of tinnitus with handicap in life; possible cause of tinnitus.

Aiming at determining the auditory thresholds, and at evaluating the intelligibility of human speech, in order that pitch and loudness matching could be eventually performed, pure tone and speech audiometry of all the subjects was obtained in an acoustic booth with the aid of the AVS 500 Audiometer. Pitches of 250, 500, 1000, 2000, 3000, 4000, 6000 and 8000 Hz (air conduction) and 500, 1000, 2000, 3000 and 4000 Hz (bone conduction) were evaluated, with the latter being performed only if the individual had an auditory threshold greater than 25 dBNA (Hearing Level). The method used to determine the auditory threshold was the descending-ascending method.

Imitanciometry was used for evaluation of the middle ear, and the compliance of the tympanic membrane and the acoustic reflexes were checked, contra- and ipsilaterally, through pitches of 500, 1000, 2000 and 4000 Hz (contralaterally), and 1000 and 2000 Hz (ipsilaterally), using the AT 235 imitanciometer.^16^

Pitch and loudness matching is used to measure the sensory characteristics of the tinnitus experience^18^; it was performed in an acoustic booth, using the same audiometer (AVS 500). This measure may have diagnostic significance; it provides a quantitative measure to monitor tinnitus deterioration or improvement; it classifies the type of tinnitus; and provides a more significant psychoacoustic score than some of the handicaps caused by tinnitus.^19^ The procedure took place as follows: it was explained to the patients that the sound that most closely resembled their tinnitus would be investigated.^20^

Sounds of different loudness and pitch were offered, and the patients indicated which one more closely resembled their tinnitus. Whenever the tinnitus was unilateral, the sound was offered to the contralateral ear. If the tinnitus was bilateral, the sound was offered to the ear with lower tinnitus loudness.^21^

First, the sound characteristic was investigated, using the stimulus that most closely resembled the tinnitus (Narrow Band Noise, White Noise, Speech Noise, Frequency Modulation, Continuous Pure Tone, or Pure Pulsed Tone); then, the pitch was analyzed: the patient chose between two different sounds, for example a 125-Hz sound, and another of 8000 Hz, and was asked “which of these sounds is closer to your tinnitus?”. The pitch was expressed in hertz (Hz), corresponding to the perception of tinnitus frequency. Next, loudness was investigated, with an increase in sound loudness given at every 1 dB. The result was expressed in dBNS (sensation level).^18^

THI is a self-report measure to quantify the impact of tinnitus on daily life, created by Newman et al. (1996).^7^ THI was validated for Brazilian Portuguese in 2006.^22^

THI was conducted as an interview, and the volunteer chose one of three possible answers to each of the 25 questions: “yes” (4 points), “no” (0 points) or “sometimes” (2 points). Each question relates to one of the domains: functional, emotional or catastrophic.^23^ The functional domain (11 items) is related to the limitations of function in mental, social/occupational, physical functioning; emotional (9 items), anger, frustration, irritability, depression; Catastrophic (5 items), despair, loss of control, inability to cope and escape, fear of severe illness.^16^

The sum of the scores obtained could thus vary from 0 to 100. According to the value obtained, the grade of handicap caused by tinnitus in each patient was classified as slight (0–16), mild (18–36), moderate (38–56), severe (58–76) or catastrophic (78–100).^7^, ^16^

VAS provides numeric estimates of tinnitus severity: patients were asked to observe the graduated scale. They were asked to score the loudness of tinnitus handicap from 0 to 10. The closer to 10, the greater the handicap referred.^7^, ^8^, ^16^

The data collected was statistically treated. For this, inferential analysis was performed: initially, a normality test, Kolmogorov–Smirnov, was performed; then, the Spearmann correlation test was established. Differences were considered significant when they presented a level of significance of 5%. Statistical analysis was performed using Statistic Package for Social Sciences (SPSS) software, version 24.

## Results

The study consisted of 148 volunteers, 35.8% male, and 64.2% female, with a mean age of 50 years (SD = 1.3). Sudden onset of tinnitus was observed in 53%, followed by gradual tinnitus in 38% of patients.

**Figure 1:**
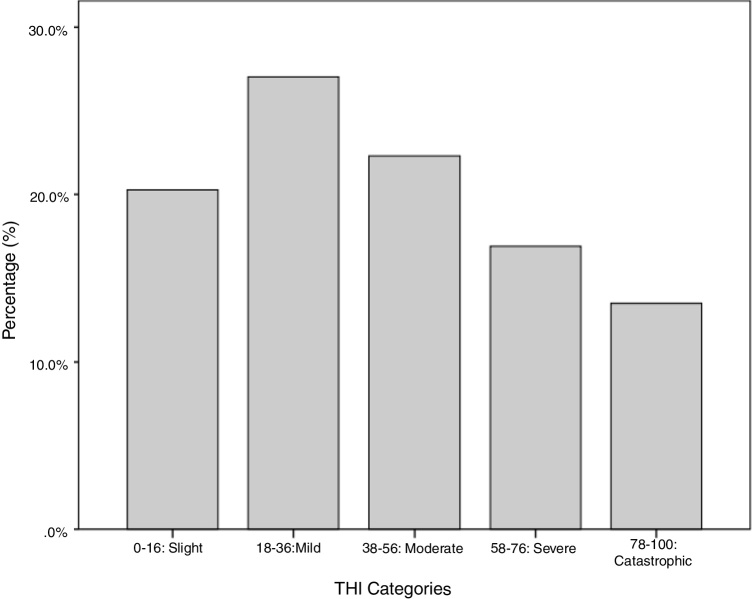
Frequency of responses of THI (tinnitus handicap inventory).

Regarding the frequency of THI responses, a higher occurrence of mild grades, followed by moderate grades, was observed (Fig. 1).

In the analysis of VAS score, an average of 6 points (SD = 2.84) was observed, with 10 being the most frequently reported by patients, followed by 4 and 8.

The mean loudness measured in the right ear was 20 dBNS (SD = 14.63) and in the left ear was 17 dBNS (SD = 14.96), the most common type of stimulus was a continuous pure tone, and peak loudness at 6000 Hz was reported by the largest number of subjects.

Regarding age and measures of tinnitus, through Spearman's correlation, small effects were observed, with positive covariance. Thus, as one value increases the other also increases. However, there was no significant difference (*p* < 0.05) (Table 1).

**Table 1:** Correlation between age and the tinnitus assessment measures.

	Spearman's correlation	*p*-value^a^
Age × THI	0.012	0.883
Age × VAS	0.062	0.451
Age × loudness matching (right ear)	0.133	0.175
Age × loudness matching (left ear)	−0.058	0.532

a *p* < 0.05 (Spearman's correlation test). THI, tinnitus handicap inventory; VAS, visual analogic scale.

Among THI and VAS measurements, a significant relationship (*p* < 0.05) with a positive covariance was observed through Spearman's correlation; that is, as one measure increases, the other increases as well. Similarly, loudness matching and VAS present a significant relationship (*p* < 0.05), but with low effect (Table 2).

**Table 2:** Correlation among tinnitus assessment measures.

	Spearman's correlation	*p*-value^a^
THI × VAS	0.570	0.001^a^
THI × loudness matching (right ear)	0.121	0.217
THI × loudness matching (left ear)	0.121	0.186
VAS × loudness matching (right ear)	0.198	0.042^a^
VAS × loudness matching (left ear)	0.248	0.006^a^

a *p* < 0.05 (Spearman's correlation test). THI, tinnitus handicap inventory; VAS, visual analogic scale.

## Discussion

The evaluative methods of tinnitus are important tools to characterize it and to document therapeutic efficacy. Currently, the most used are: THI, VAS, and pitch and loudness matching. The standardization of these methods and the identification of the relationship among them is extremely important.

In the present study, when analyzing the frequency of THI responses, a higher occurrence of mild, followed by moderate grades, was observed. The same result was found in the literature,^24^ in which 67%–70% of 129 tinnitus patients felt no handicap from tinnitus, or classified it as mild. However, studies have also observed the presence of severe or catastrophic grade,^24^ and moderate or severe.^25^, ^26^

In the analysis of the VAS score, when characterizing handicap caused by tinnitus, an average of 6 points was observed. The most frequently reported score mentioned by volunteers was 10, which was the maximum score, and indicated a high discomfort due to the symptom. The literature has indicated a mean that is close to this research, being 7 for patients who were assiduously followed by a clinic for 3 months, and 6 for a group that was not so assiduous, both before treatment for tinnitus.^25^, ^26^

It is understood that the question of tinnitus severity and degree of handicap depends on some determining factors, such as: psychological, cognitive and personality traits.^27^ And the higher the degree of THI, the more the individual affected with tinnitus is sensitive to other emotional and health issues, such as anxiety and sleep disorders.^28^ In the present study, mild grade findings may be related to low anxiety common to all volunteers. This implies that the severity of the symptom may actually be linked to, or aggravated by, psychological issues. This was not the main objective of the study, but it is a point to be taken into consideration in the evaluation of patients complaining of tinnitus. Mainly because there is an emotional domain in THI that should be carefully evaluated in these patients.

Regarding the psychoacoustic measures of tinnitus, an average of 20 and 17 dBNS of loudness was found for right and left ears, respectively. The most frequent pitch sensation was acute, and the type of tinnitus stimulus was pure continuous tone. Such measures are consistent with other studies.^29^, ^30^ However, there is no agreement in the literature in this regard, since no differences in the psychoacoustic properties of tinnitus (loudness, pitch, and minimum level for its suppression) have been demonstrated among tinnitus patients who suffer with it and those who do not suffer with it.^31^

There was no significant relationship between age and measures of tinnitus (THI, VAS, and pitch and loudness matching). Interestingly, another study reports that younger individuals who had tinnitus had significantly greater stress, anxiety, and distress, classifying tinnitus as catastrophic. That is, young people are less tolerable to it than older individuals.^24^ However, a study^32^ with a mean age similar to that in the present study found no significant correlation of tinnitus handicap with the variables gender, age and degree of hearing loss.

A strong positive correlation was observed between THI and VAS measurements, because when one value increases, the other increases. These findings were also observed in the researched literature.^8^ Even though THI is a more comprehensive evaluation, the use of VAS is simpler and easier for patients to assimilate, and it is equally reliable.^7^

There was a significant relationship between pitch and loudness matching and VAS, but with a small effect. These data contrast with other studies that did not show a significant correlation between psychoacoustic tinnitus measurements and subjective assessment of tinnitus severity (VAS).^33^

There was no relation between THI and pitch and loudness matching measurements. This was also reported by another study, which indicated that any psychoacoustic characteristic of tinnitus, such as loudness or pitch, are not related to any relevant disadvantage to tinnitus.^24^ Diverging from the findings,^15^ they found a weak but significant correlation between the degree of handicap of THI and tinnitus loudness matching.^14^, ^27^

These findings, mainly of the degree of handicap and the sensation of tinnitus loudness, are intriguing, since there is no significant relation between them. Many practitioners in clinical settings have found patients who have mild THI, low loudness sensation level, VAS at peak score, and are unable to carry out their daily activities normally., Other factors, such as the emotional ones, probably should be investigated concomitantly.

However, the point is that, to date, the evaluative methods that are most used are THI, VAS, and pitch and loudness matching. Therefore, these should be intensively studied in order to find scientific evidence to help clinical practice and evaluation of the symptom.

In scientific research, it is important to quantify tinnitus both for its subjective characteristics (pitch and loudness) and its impact, since both approaches are complementary and consider different aspects of tinnitus.^14^ Thus, the selection of one of three evaluative methods for tinnitus investigation provides different dimensions of tinnitus, contributing to a broader view of the handicap generated. Also, the tests are related to each other when their values are compared in the studied group. Therefore, the use of more than one method to prove the results is important, since the study materials complement each other.

## Conclusion

The present study showed a significant positive correlation between the values measured by THI and VAS (handicap), and between loudness matching and VAS in the evaluation of tinnitus.

## Conflicts of interest

The authors declare no conflicts of interest.
